# Understory Plant Community Composition Is Associated with Fine-Scale Above- and Below-Ground Resource Heterogeneity in Mature Lodgepole Pine (*Pinus contorta*) Forests

**DOI:** 10.1371/journal.pone.0151436

**Published:** 2016-03-14

**Authors:** Anne C. S. McIntosh, S. Ellen Macdonald, Sylvie A. Quideau

**Affiliations:** Department of Renewable Resources, University of Alberta, Edmonton, Alberta, Canada; University of Saskatchewan, CANADA

## Abstract

Understory plant communities play critical ecological roles in forest ecosystems. Both above- and below-ground ecosystem properties and processes influence these communities but relatively little is known about such effects at fine (i.e., one to several meters within-stand) scales, particularly for forests in which the canopy is dominated by a single species. An improved understanding of these effects is critical for understanding how understory biodiversity is regulated in such forests and for anticipating impacts of changing disturbance regimes. Our primary objective was to examine the patterns of fine-scale variation in understory plant communities and their relationships to above- and below-ground resource and environmental heterogeneity within mature lodgepole pine forests. We assessed composition and diversity of understory vegetation in relation to heterogeneity of both the above-ground (canopy tree density, canopy and tall shrub basal area and cover, downed wood biomass, litter cover) and below-ground (soil nutrient availability, decomposition, forest floor thickness, pH, and phospholipid fatty acids (PLFAs) and multiple carbon-source substrate-induced respiration (MSIR) of the forest floor microbial community) environment. There was notable variation in fine-scale plant community composition; cluster and indicator species analyses of the 24 most commonly occurring understory species distinguished four assemblages, one for which a pioneer forb species had the highest cover levels, and three others that were characterized by different bryophyte species having the highest cover. Constrained ordination (distance-based redundancy analysis) showed that two above-ground (mean tree diameter, litter cover) and eight below-ground (forest floor pH, plant available boron, microbial community composition and function as indicated by MSIR and PLFAs) properties were associated with variation in understory plant community composition. These results provide novel insights into the important ecological associations between understory plant community composition and heterogeneity in ecosystem properties and processes within forests dominated by a single canopy species.

## Introduction

Understory vegetation is an integral component of the ecological functioning and biodiversity of forests [1]. The understory plant community plays important roles in influencing both community and ecosystem properties above- and below-ground [2, 3]. Persistent dense understories can be an important filter in determining the future successional trajectory of forests by reducing or delaying tree regeneration and growth [4, 5]. The forest understory can also play an important role as a food source and as habitat for a variety of other biota [6, 7].

The composition and biodiversity of these ecologically important understory plant communities is affected by both above- and below-ground factors, with complex interactions among them [8]. The primary focus of many studies has been on examining the effects of overstory composition on understory plant communities. For example, studies have demonstrated that forest canopy trees can affect the understory plant community through above-ground (e.g., light availability, litter deposition) and/or below-ground (e.g., soil pH, nutrient and moisture availability) resources ([9–11], [3], reviewed by [12–14]). On the other hand, understory communities are not always related to forest canopy type [15], suggesting that other variables, including below-ground factors unrelated to canopy composition, may also be important in structuring understory plant community composition. Below-ground microbial communities actively contribute to ecosystem functions that include litter decomposition [16], plant productivity [17], and plant nutrition through mycorrhizal relationships [18]. Symbiotic relationships between bacteria and plants are also important; for example, in nitrogen-limited forests, nitrogen fixation in root nodules may contribute a significant portion of the plant available nitrogen to understory species [19]. Thus, we expect that the soil microbial community could have an important influence on understory plant communities. Yet, explicit relationships between understory plant communities and below-ground resources remain underappreciated and understudied in most plant community ecological studies [20].

Thus, there is still considerable uncertainty about which below-ground properties or processes are important in structuring understory plant communities and their relative importance compared to above-ground properties and processes. A recent review suggested that at the stand level (i.e., scale of hectares) resource quantity may be the primary driver of understory species diversity, whereas resource heterogeneity may be the primary driver at broader scales (i.e., scale of square kms). However, there was no consideration of the fine (micro-habitat) scale (i.e., one to several m^2^ within an individual forest stand) [21].

Identifying the factors controlling compositional heterogeneity within environments that at first appear homogenous is a great challenge in ecology. In forests with a monodominant canopy the vegetation biodiversity is found in the understory; these forests provide a valuable test system for evaluating the relative importance of various above- and below-ground properties and processes in structuring plant community composition at a fine scale. Fine-scale spatial heterogeneity in environmental conditions and resources is likely to be lower within such forests, as compared to those with a diversity of tree species forming the canopy. Still we expect some level of micro-habitat variation (e.g., due to small gap formation, micro-topographic variation, inputs of coarse woody material) that will be associated with fine-scale variability in ecosystem properties and processes that influence understory plant community composition. Partitioning of understory species across these heterogeneous conditions within superficially homogeneous stands could be a key process driving understory biodiversity in such forests. However, studies examining patterns in understory plant community composition at the within-stand scale have focused on variation among patches (i.e. tens of m^2^ scale) of differing canopy species or types e.g., [10]; only a few studies have examined fine-scale variation [22].

Insight into factors regulating biodiversity in forests dominated by a single canopy species is critical for ecologically-sustainable management, particularly as forest composition and structure are affected by forest management and changing disturbance regimes. Our objective was to examine the patterns of fine-scale variation in understory plant communities and their relationships to above- and below-ground resource and environmental heterogeneity within mature lodgepole pine (*Pinus contorta* Dougl. ex Loud.) forests. While previous below-ground studies have focused primarily on relationships between plants and soil chemistry [22], we expanded our focus to include below-ground microbial community structure, using phospholipid fatty acid (PLFA) analysis [23] to assess microbial biomass and community structure and multiple carbon source substrate-induced respiration (MSIR) [24] to produce a physiological profile of the microbial community (i.e., function). We hypothesized that, even in these monodominant canopy forests, understory plant community composition would be associated with fine-scale environmental and resource heterogeneity (e.g., light, nutrient levels). Given the importance of light for understory species, and their varied distribution across a range of tolerances to light/shade, we expected variation in light levels to be an important above-ground source of heterogeneity as has been demonstrated in other studies [16]. We expected that in these N-limited ecosystems, fine-scale variation in plant available nitrogen would also contribute to fine-scale variation in plant community types. Further, given the important ecological roles of soil microbes and their contributions to below-ground variation, we predicted that below-ground microbial properties would be prominent among the variables associated with patterns of variation in plant community composition.

## Materials and Methods

### Study area

The study area was located in the Upper Foothills Natural Subregion of Alberta, Canada [25] in lodgepole pine forests near Robb (53.2336N, 116.9745W). This region is characterized by pure lodgepole pine forests, along with mixed conifer forests of white spruce (*Picea glauca* (Moench) Voss) and subalpine fir (*Abies lasiocarpa* (Hook.) Nutt.). Lodgepole pine is a coniferous tree with a wide climatic and geographical range in North America; it forms forest stands that are highly valued for timber, wildlife habitat and recreational use. Across a wide portion of its range the landscape is characterized by forest stands heavily dominated by just this single canopy species. Stand ages in this region are generally younger than 100–120 years old, reflecting the regional disturbance regime of relatively frequent stand-initiating wildfire [26]. This relatively frequent disturbance is accompanied by reorganization of the understory plant community [27]. The study area experiences a temperate continental climate where mean daily maximum air temperatures during the growing season range from 16.2°C in May, to 20.6°C in August (30 year climate normal 1971–2000). Mean monthly precipitation from May to August ranges from 57.9 to 82.2 mm, with a mean annual precipitation of 562.4 mm (30 year climate normal 1971–2000).

A total of three forest study units ranging in size from 4.8–8.8 ha were sampled during the summer growing season of 2008 (Table 1). We were given permission by project collaborators West Fraser Timber Company to sample these study units, which were located in their Forest Management Agreement Area on public (crown) land. The study units we selected were relatively flat topographically, were similar to one another and covered by mature (~ 110–120 yrs) lodgepole pine forest representative of the dominant forest cover type in the region. The study units were classified as ecosite UF e1.1 –Pl/green alder/feather moss [26], and were located on brunisolic gray luvisolic soils [28]. The overstory (i.e., trees with dbh > 5 cm) was predominantly lodgepole pine; there were a very few white and black spruce (*Picea mariana* (Mill.)), trembling aspen (*Populus tremuloides* Michx.) and balsam fir (*Abies balsamea* (L.) Mill) in the lower canopy (i.e., trees > 5 cm dbh and ~ 1.3–7 m ht). The understory (i.e., all of the vascular and non-vascular plants located below the forest canopy including seedlings (< 1.3 m ht) and saplings (i.e., > 1.3 m ht but < 5 cm dbh)) was dominated by feather mosses, including *Pleurozium schreberi* (Brid.) Mitt., *Ptilium crista-castrensis* (Hedw.) De Not. and *Hylocomium splendens* (Hedw.) Schimp. and the hair cap moss *Polytrichum commune* Hedw. Common forbs included *Cornus canadensis* L. and *Linnea borealis* L. and common small shrubs included *Rosa acicularis* Lindl. and *Vaccinium myrtilloides* Michx.; *Alnus crispa* (Aiton) Pursh was the dominant tall shrub and *Calamagrostis* spp. were the most common graminoids. Notably, advance tree regeneration was absent or present in very low numbers (i.e., < 10 seedlings or saplings– ha^-1^; [29]). There was no evidence of current or prior mountain pine beetle (*Dendroctonus ponderosae* Hopkins (Coleoptera: Curculionidae, Scolytinae)) attack, which is a novel disturbance agent in the region, in these stands at the time of the study.

**Table 1:** Summary of site characteristics. Given are the locations and mean values for each of the three lodgepole pine forest study units. Basal area and density were calculated for each sample point and then averaged within each study unit, whereas mean dbh was calculated using all trees within a study unit; the minimum and maximum values across sampling points within each study unit (n = 36) are in parentheses.

Study Unit	Latitude/Longitude	Basal area (m^2^ ha^-1^)	Density (trees ha^-1^)	Dbh (cm)	Canopy cover (%)
1	53.2248W/116.8094N	39.6 (26.7–56.2)	1420 (950–1900)	18.3 (5.0–34.7)	63.9 (56.2–86.9)
2	53.24129W/116.8288N	37.3 (21.6–55.1)	978 (550–1350)	21.5 (6.6–43.3)	59.2 (51.4–70.7)
3	53.22647W/116.8212N	40.3 (27.1–54.0)	1182 (450–1850)	20.1 (8.0–38.3)	62.1 (54.9–77.4)

Within each study unit we established four 0.48 ha (60-m x 80-m) plots. Each plot was surrounded by a minimum of 20-m (~ one tree height) of pine forest in order to minimize edge effects. Plots within a study unit were placed as close together as possible within the constraint of ensuring uniform overstory stand conditions within each plot (i.e., similar density and size distribution of canopy trees). Within each plot we established nine systematically-located sample points that were used as the center/starting-points for sampling the overstory, downed wood, understory and below-ground (n = 3 study units * 4 plots * 9 sample points = 108 sampling points). These sample points were located 20–30 m apart from one another to reduce spatial auto-correlation as much as possible.

### Data collection

#### Above-ground sampling

The overstory canopy plant community was sampled in 8-m fixed-radius (0.02 ha) circular subplots centered at each of the sample points. Standard forest mensuration data were collected for all trees (i.e., with dbh ≥ 5 cm and ht > 1.3 m) within each subplot (i.e., live/dead status, species and dbh). These data were used to calculate basal area and stem density, broken out both by live/dead status and by species.

To estimate canopy cover, hemispherical photographs were taken in mid-July at 1.4 m height at each sample point using a digital Nikon Coolpix 4500 with FC-E8 fisheye lens. We calculated gap fraction of images using SLIM (Spot Light Intercept Model v. 3.01) and subtracted gap fraction from 100 to estimate canopy cover (see [30] for details).

The downed woody material (DWM) was measured at each sample point using the line intersect method [31–34] along 8-m transects that started at each sampling point. Total biomass (Mg ha^-1^) and biomass estimates for each of six diameter size classes (0–0.5 cm, 0.5–1.0 cm, 1–3 cm, 3–5 cm, 5–7 cm and > 7 cm) were calculated using the equation and coefficients for Central Alberta Foothills lodgepole pine stands ([35, 36]; see [30] for detailed description). Percent cover of DWM was estimated during assessment of understory communities (see below).

We sampled the understory plant community (i.e., forest floor mosses, forest floor lichens, forbs, graminoids, shrubs also including tree seedlings and tall shrubs < 1.3 m tall—see S1 Table for detailed list) within 1-m^2^ quadrats located at each sample point. Percent cover (0–100) of each species/taxa was estimated. For species not identified in the field, voucher specimens were collected for identification through comparison with University of Alberta herbarium samples. Nomenclature follows the USDA Plants database (http://plants.usda.gov/). Cover estimates were also recorded for litter, tree/snag boles, downed woody material (diameter ≥ 3 cm), exposed mineral soil and rock. We measured tall shrubs and saplings (i.e., > 1.3 m ht and < 5 cm dbh, e.g., *Alnus crispa*) in 4-m radius circular subplots centered at each of the sampling points. To estimate basal area of tall shrubs and saplings within the plots, we measured the stem basal diameters for shrubs and saplings rooted within the subplot and for shrubs that weren’t rooted in the subplot but had canopy overhanging the subplot.

#### Below-ground sampling and analysis

The thickness of the forest floor (excluding the recent litter fall, or L layer, but including both Fibric and Humic layers—i.e., F/H, mm) was measured in each of the four corners of each understory quadrat.

We installed Plant Root Simulator (PRS) probe ion exchange membranes (Western Ag Innovations, Inc., Saskatoon, SK, Canada) to measure soil nutrient availability. The anion exchange PRS^™^-probes simultaneously adsorbed all nutrient anions, including NO_3_^-^, PO_4_^3-^ and SO_4_^2-^. Cation exchange PRS^™^-probes simultaneously adsorbed nutrient cations such as B^+^, NH_4_^+,^ K^+^, Ca^2+^ and Mg^2+^. A chelating pre-treatment of the anion PRS^™^-probe also permitted the adsorption of micronutrient metals such as Cu^2+^, Fe^2+^, Mn^2+^ and Zn^2+^. We installed four pairs (pair = 1 cation and 1 anion exchange membrane) of PRS probes vertically at the four corners of each of the understory quadrats. The top of the ion exchange membrane was placed at the interface between the forest floor and mineral soil and measured exchange to a depth of ~6 cm (17.5 cm^2^ of absorbing surface area). Probes were installed for the duration of the growing season (mid-June to mid-September 2008). After probes were removed at the end of the growing season, they were cleaned with deionized water and shipped to Western Ag for analysis; the four probe pairs from individual quadrats were pooled prior to elution and analysis. NO_3_-N and NH_4_-N were analyzed colorimetrically using an automated flow injection analysis system, while all remaining nutrient ion contents in the eluate were measured using inductively-coupled plasma spectrometry (www.westernag.ca). For most nutrients, the few samples for which the measured value for a nutrient was below the minimum detection limit (MDL) were still included in analysis using the values measured for them because censoring data below MDL can bias your dataset (Western Ag, personal communication). For four elements (Cd, Cu, NO_3_^-^ and Pb), we did not analyze the data because their calculated nutrient supply rates were predominately below the minimum detection limits.

Decomposition was measured at each of the sampling points using nylon mesh bags (1.5 mm x 1.5 mm mesh size) with four 90-mm diameter Whatman cellulose filter papers buried at the forest floor-mineral soil interface for the growing season (same time span as PRS probes). Filter papers were oven dried for 1 day (pre-burial) and 3 days (post-burial) at 70°C and weighed before and after being buried. Decomposition was calculated as 100 minus the percentage of original filter paper biomass remaining at removal (i.e., percent of filter paper lost over the duration of the growing season burial period).

Forest floor samples (i.e., the entire thickness of the combined F and H layers) were collected from the four corners of each understory quadrat using aseptic techniques; the four samples per quadrat were combined to form a single homogenous sample (~ 50 g) per quadrat. These were then divided into portions to be used for pH, MSIR, and PLFA analysis. Samples for MSIR and pH were sieved (4 mm) and kept refrigerated (4°C) in plastic bags prior to analysis. PLFA samples were stored at -86°C and then freeze-dried prior to PLFA extraction.

Forest floor pH was measured potentiometrically in a saturated paste in equilibrium with a soil suspension of a 1:4 soil:liquid mixture. We used 0.01 M CaCl_2_ in place of water following the instructions for measuring pH of field-moist organic samples as described in [37].

Functional composition of microbial communities relates to their activity, particularly in the carbon cycle. MicroResp^™^ multiple carbon source substrate-induced respiration (MSIR) offers a convenient, rapid and sensitive method for the determination of the microbial community-level physiological profile for each forest floor sample using a ‘whole soil’ technique that uses the same 96-well microtitre plate format that Biolog (Biolog Inc.) does [24, 38]. Detailed methods are described in [39], but to briefly summarize, we used detection agar plates containing a gel-based bicarbonate buffer with indicator dye to measure pH change within the gel resulting from carbon dioxide evolved from the soil during an incubation period of 6 hours in the dark for field moist sieved forest floor samples that were exposed to a set of carbon substrates (30 μl of each substrate was dispensed to a deep well) [40]. Fifteen substrates commonly used in carbon MSIR analysis and thought to be associated with plant root exudates [41, 42] were used: five amino acids (L-alanine, L-arginine, glutamine, L-lysine, γ aminobutyric acid), six carbohydrates (n-acetyl glucosamine, L-(+)-arabinose, D-(+)-galactose, glucose, mannose, trehalose), four carboxylic acids (citric acid, L-malic acid, oxalic acid, 3,4-dihydroxybenzoic acid) and water as a control to measure basal respiration. The colour change in the detection plate was then read on a standard laboratory microplate reader (detection plate read before and after 6 hrs of incubation, absorbance = 570 nm) and respiration rates were calculated (μg CO_2_-C g^-1^ hr^-1^). A maximum of 16 samples could be analyzed in a day, so samples were randomly selected each day to reduce bias associated with differences in time since collection and all analyses were completed within two weeks of sample collection. One sample had five carbon substrate respiration rates below basal respiration and was excluded from analysis. To measure catabolic evenness, we used the Simpson-Yule index (1/∑p_i_^2^, [43]), where p_i_ is the respiration response for an individual substrate as a proportion of total respiration rates from all substrates for a given forest floor sample [44].

Microbial phospholipid fatty acid (PLFA) analysis produces a lipid profile of microbial communities. To measure microbial PLFA structure we transferred 0.30 g of each freeze dried forest floor sample to a muffled test tube and then analyzed each of them following the methods described in [45]. To summarize, we analyzed forest floor samples by extraction with a single-phase chloroform mixture, lipid fractionation on a solid-phase-extraction Si column and then subjected them to a mild methanolysis using a modified Bligh and Dyer extraction [46–48]. The resulting fatty acid methyl esters were then analyzed (see [30] for details). Fatty acids were designated X:YωZ, where X represents the number of carbon atoms, Y represents the number of double bonds and Z indicates the position of the first double bond from the aliphatic (ω) end of the molecule. The suffixes c and t indicate cis and trans geometric isomers. The prefixes ‘a’ and ‘i’ refer to anteiso and iso branching and Me and OH specify methyl groups and hydroxyl groups, respectively. PLFAs that were present in 5% or less of the samples were excluded from analysis. PLFAs for 16:1ω9c and 16:1ω11c, and 18:2 ω6,9c and 18:0a were combined for analysis as they could not be distinguished by the GC. We excluded two samples with < 85% peak matching from analysis. There were a total of 54 PLFAs included in the final analysis and these were also summed to provide a measure of total PLFA biomass (nmol g^-1^ forest floor). PLFAs that have been previously identified as associated with soil microorganisms were combined into PLFA biomarker groups for actinomycetes, arbuscular mycorrhizae, bacteria, and fungi (Table 2). The ratio of fungal to bacterial PLFAs was used to estimate the relative contributions of fungi and bacteria. Aside from the measurement of total biomass of PLFAs and the biomass for biomarker groups, all measured PLFAs were expressed on a mol% basis to standardize for differences in the total amount of forest floor PLFAs among samples.

**Table 2:** Phospholipid Fatty Acids (PLFAs) that have been previously identified as biomarkers of given microbial groups.

PLFAs	Biomarker Group	References
10me16:0, 10me17:0 and 10me18:0	Actinomycetes	[49, 50]
16:1ω5c	Arbuscular mycorrhizae	[51, 52]
10:0 3OH, 12:0, 12:0 2OH, 12:0 3OH, 14:0, i14:0, 15:0, a15:0, i15:0, i16:0, i17:0, a17:0, 17:0, cy17:0, 18:1 ω5c, 18:1ω7c	Bacteria	[51, 53, 54, 55], [56]
18:1ω9c, 20:1ω9c, 18:3ω6c	Fungi	[55, 57]

To provide a standardized measure of specific respiration rates of the microbial community we also calculated the microbial metabolic quotient for each sample as the ratio of soil basal respiration (i.e. MSIR with water as the substrate) to microbial biomass (i.e., the total PLFA biomass) (qCO_2_ -[58]).

### Statistical Analyses

The minimal dataset underlying the findings of the study that were used in the analysis in this study are included as a MS Excel file in the supplementary materials (S1 File).

Twenty four understory species/taxa occurred in 5% or more of quadrats while a further 16 understory species occurred in < 5% of quadrats (see S1 Table). The understory species richness we observed is similar to richness measured in previous lodgepole pine studies [59]. For data analysis we included only understory species/taxa that were found in > 5% of quadrats because we considered that including infrequent species would add noise to the dataset and hamper our ability to distinguish patterns and associations [60].

To examine patterns of variation in understory assemblages and to group the quadrats into plant community types/assemblages we used hierarchical, agglomerative clustering on cover data for the 24 species/taxa. Cluster analysis is a powerful tool to interpret vegetation patterns [61] and we used a flexible beta linkage method, which uses an agglomerative hierarchical algorithm where the user can specify the beta parameter [62], with β = -0.25 and Sørenson’s distance measure. Because we did not have *a priori* types, Indicator Species Analysis (ISA; [63]) was used to prune the dendrogram of the cluster analysis by comparing different numbers of clusters (i.e., 1 to 12) and then selecting the number of clusters with the highest number of significant indicator species and the lowest average P-value [60]. Once the number of clusters had been finalized, ISA using Monte Carlo permutations (n = 5000) was used to determine which species were significant (alpha = 0.05) indicators for each cluster (i.e., plant community type). Cluster analysis and ISA were conducted using PC-ORD (Version 5 MjM Software Design, Gleneden Beach, OR).

To examine associations of understory community composition with above-and below-ground properties we used constrained ordination (Distance-based redundancy analysis (db-RDA) (see [64–66, 30] for details). We used the Bray-Curtis distance measure and excluded negative eigenvalues in our PCoA. Environmental variables were tested for inclusion in the db-RDA with forward step-wise selection and testing for significance (alpha = 0.05) by 499 Monte Carlo permutations, blocked by Study Unit. A final db-RDA including only the significant variables was run to test the significance of the first and all canonical axes. Canoco for Windows Version 4.56 [66] was used for analyses. A list of all of the environmental variables that were used in the multivariate statistical analysis can be found in S2 Table.

For the resource and environmental variables that were significant in the db-RDA, we used analysis of variance (ANOVA: Proc Mixed procedure in the SAS statistical software package (SAS Institute Inc., Version 9.2 (32-bit), Cary, NC, USA: SAS Institute Inc. 2008) to compare the mean values among the four plant community types (as identified by the cluster analysis). Plot within study unit was included as a random term while plant community type was the fixed effect. We first determined whether each variable met the assumptions for ANOVA using analysis of residuals and normal probability plots; we transformed response variables when necessary. When significant differences were detected, we used post-hoc linear contrasts for pairwise comparisons among plant community types with Bonferroni-adjustment of P-values (family-wise alpha = 0.05) (Proc Mixed, SAS v 9.2).

Four of the 108 quadrats were excluded from analysis because of low PLFA peak matching, low respiration rates, or based on outlier analysis of environmental variables used in the ordination.

## Results

The cluster analysis distinguished four assemblages, i.e., plant community types (see also S1 and S2 Figs for more details). Community type 1 was characterized by two mosses and two dwarf/trailing woody vascular plants; Type 2 by two mosses, one grass and one shrub; Type 3 by a single feather moss species; and Type 4 by three forbs and two shrubs (Table 3).

**Table 3:** Results of indicator species analysis. Species that had an indicator value >20 and were significant at α = 0.05 are listed in order by descending indicator value within each plant community type. Mean cover values (± SE) for each of the indicator species for all four plant community types are also provided, highlighted in bold is the cover values for the plant community type a species was an indicator for.

Community type	N*	Species	Indicator value	P	Cover (± SE)			
					Type 1	Type 2	Type 3	Type 4
1	18	*Hylocomium splendens*	54.5	0.0002	**21.7 (5.1)**	4.3 (1.0)	2.7 (0.6)	6.7 (2.3)
		*Linnea borealis*	40.2	0.0012	**15.9 (2.8)**	10.0 (1.9)	9.1 (1.6)	4.5 (0.9)
		*Cornus canadensis*	34.7	0.02	**17.0 (3.7)**	13.4 (1.6)	9.6 (1.5)	9.0 (0.9)
		*Dicranum polysetum*	26.8	0.01	**2.0 (0.7)**	0.4 (0.2)	0.8 (0.3)	0.6 (0.4)
2	25	*Ptilium crista-castrensis*	61.0	0.0002	3.6 (1.1)	**25.6 (4.1)**	1.0 (0.3)	8.4 (1.4)
		*Polytrichum commune*	40.2	0.003	0.8 (0.4)	**10.4 (3.5)**	1.9 (1.0)	4.5 (1.6)
		*Calamagrostis* spp.†	38.7	0.001	3.1 (1.6)	**15.8 (4.0)**	3.5 (1.4)	3.8 (1.2)
		*Rhododendron groenlandicum*	22.3	0.01	0.3 (0.3)	**11.7 (5.8)**	1.2 (1.0)	1.5 (1.0)
3	31	*Pleurozium schreberi*	63.8	0.0002	8.6 (2.4)	14.9 (3.4)	**54.1 (3.9)**	7.1 (2.0)
4	30	*Chamerion angustifolium*	80.2	0.0002	0.6 (0.3)	2.2 (0.8)	2.1 (0.9)	**19.7 (2.4)**
		*Lycopodium annotinum*	42.6	0.0002	0.2 (0.2)	2.7 (1.0)	0.1 (0.1)	**12.1 (3.0)**
		*Aralia nudicaulis*	39.0	0.0002	1.1 (0.9)	3.9 (1.8)	1.5 (0.8)	**10.3 (2.2)**
		*Rosa acicularis*	34.7	0.005	4.1 (1.3)	4.2 (1.0)	3.1 (0.8)	**6.7 (0.9)**
		*Rubus pubescens*	33.3	0.0008	0.5 (0.4)	0.6 (0.4)	0.3 (0.2)	**2.8 (1.0)**

* This is the number of quadrats that were of that plant community type.

^†^ Much of the *Calamagrostis* grass did not flower in our sites, and therefore we have referred to this graminoid as *Calamagrostis* spp.

**Fig 1:**
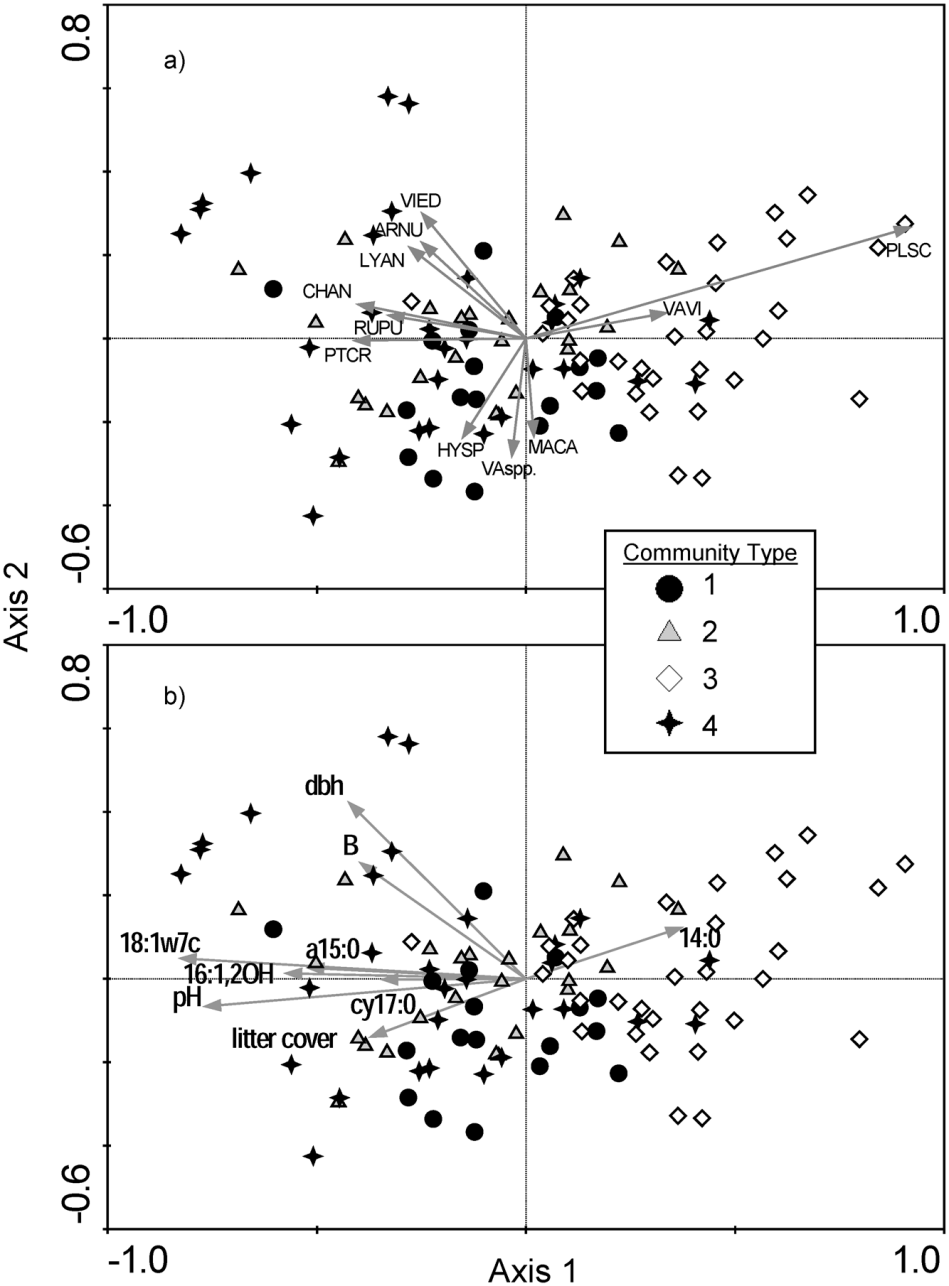
Results of distance-based redundancy analysis of understory plant community composition delineated by the four plant community types identified by hierarchical cluster analysis: a) Uppercase four letter codes indicate the locations of plant species which had a Pearson correlation coefficient > 0.3 (see S1 Table for description of species codes), and b) the direction and length of the vector for environmental variables (see Table 5 for details of the abbreviated vector labels) reflects the strength of correlation with the first two axes for variables that had a Pearson correlation coefficient > 0.3 for either of the first two axes. Each symbol is a quadrat, which is coded by plant community type. To improve readability the environmental and species scores were scaled up 3.3 and 2.5 times, respectively, to those of the sample scores and some species points were moved slightly from their original location.

The constrained ordination illustrated separation among the four fine-scale plant community types, which species contributed to that separation, and the relationship of the environmental variables to variation in understory composition. The first four db-RDA axes, which were all significant, accounted for 84.4% of the species-environment relationship (Table 4), and quadrats of the four plant community types separated along the first two db-RDA axes (Fig 1). Two above- and eight below-ground variables were significantly correlated with understory plant community composition along the four axes and collectively explained 24.1% of the variation in the species data (Table 4, Fig 1b). Forest floor pH and the bacterial PLFAs 18:1ω7c, a15:0 and 14:0, and PLFA 16:1 2OH were most strongly correlated with Axis 1, mean dbh of overstory trees and available boron were the variables most highly correlated with Axis 2; litter cover and the bacterial PLFA cy17:0, and respiration of malic acid were most strongly correlated with Axes 3 and 4, respectively (Table 4). The locations of quadrats of the different plant community types and species in ordination space were consistent with the indicator species analysis, illustrating compositional differences among the four plant community types (Fig 1).

**Table 4:** Results of distance-based redundancy analysis. The trace value (sum of all the canonical eigenvalues) and the eigenvalues of the first four axes are presented, along with the species-environment correlations, and the cumulative percentage of the variance explained for species and species-environment. Inter-set correlations (Pearson) of significant above- and below-ground variables from the db-RDA step-wise forward selection (see Table 5 for description of variables), presented in order by their correlations (from high to low) with the first axis. The inter-set correlation values for the axis where the correlation was strongest are highlighted in bold.

	Axis 1	Axis 2	Axis 3	Axis 4
Trace: 0.262				
Eigenvalues*	0.133	0.037	0.032	0.019
Species-environment correlations	0.842	0.701	0.714	0.632
Cumulative percentage variance:				
Species data	14.5	18.6	22.1	24.1
Species-environment relation	50.9	65.2	77.3	84.4
Inter-set correlations:				
PLFA 18:1ω7c (bacteria)	**-0.626**	0.058	0.004	-0.146
pH	**-0.560**	-0.077	-0.263	0.0091
PLFA 16:1 2OH	**-0.447**	0.016	-0.054	-0.054
PLFA a15:0 (bacteria)	**-0.376**	0.030	0.335	0.242
Dbh	-0.351	**0.550**	-0.143	-0.078
B	-0.284	**0.313**	-0.069	0.183
PLFA cy17:0 (bacteria)	-0.275	-0.003	**-0.357**	0.013
PLFA 14:0 (bacteria)	**0.262**	0.136	-0.0004	0.061
Litter	-0.259	-0.153	**-0.438**	0.372
Malic acid	0.115	0.211	-0.164	**0.287**

* Axis 1 and all axes combined were significant at P = 0.02

There were significant differences in the measured environmental variables among the four plant community types; these reflect the patterns observed in the db-RDA with one above-ground and five below-ground variables being significant (Table 5). The only above-ground variable that differed among types was mean dbh of trees; plant community type 4 had larger diameter trees than types 1 and 3, while type 2 was intermediate. Forest floor pH differentiated community type 4 from community types 2 and 3, with intermediate levels in quadrats of community type 1. The bacterial PLFA 18:1ω7c separated community type 3 from the remaining community assemblages. PLFA 16:1 2OH (which has not yet been identified as a biomarker for any microbial groups) distinguished community type 3 from types 2 and 4, with intermediate levels in community type 1. Community type 3 differed with type 2 for bacterial PLFA a15:0, with intermediate levels in community types 1 and 4. Bacterial PLFA cy17:0 distinguished community type 3 and community type 1, but not from community types 2 and 4. There were no significant differences among plant community types for the bacterial PLFA 14:0, plant available boron, litter cover, or malic acid respiration (Table 5).

**Table 5:** The mean values (± SE) for the above- and below-ground variables that were significant in the distance-based redundancy analysis ordination for each of the four plant community types (see Table 4). More information on biomarker phospholipid fatty acids is located in Table 2. Different lower case letters (a, b, c) after mean values indicate significant differences (alpha = 0.05) for individual variables among plant community types based on one-way ANOVAs.

Variable code	Description*	Units	Plant Community Type			
			1	2	3	4
Above-ground variables						
Dbh	Mean stem diameter	cm	18.8 (0.3)b	19.8 (0.5)ab	19.3 (0.3)b	22.1 (0.06)a
Litter	Cover of litter	%	55.6 (4.8)	50.8 (4.4)	42.3 (2.1)	53.0 (4.4)
Below-ground variables						
pH	Forest floor pH	n/a	3.69 (0.04)ab	3.52 (0.04)bc	3.38 (0.03)c	3.80 (0.06)a
18:1ω7c	Phospholipid fatty acid	mol%	9.41 (0.27)b	9.37 (0.40)b	7.88 (0.27)a	10.64 (0.26)b
a15:0	Phospholipid fatty acid	mol%	2.26 (0.08)ab	2.46 (0.07)a	2.20 (0.06)b	2.45 (0.06)ab
16:1 2OH	Phospholipid fatty acid	mol%	0.20 (0.05)ab	0.23 (0.04)b	0.10 (0.02)a	0.31 (0.03)b
B	Plant available boron	μg -10 cm^2^ –summer burial^-1^	1.01 (0.11)	1.00 (0.09)	0.75 (0.07)	0.96 (0.09)
cy17:0	Phospholipid fatty acid	mol%	1.99 (0.06)a	1.86 (0.07)ab	1.81 (0.05)b	2.07 (0.05)ab
14:0	Phospholipid fatty acid	mol%	1.55 (0.10)	1.51 (0.05)	1.63 (0.05)	1.50 (0.04)
Malic acid	Respiration rate	μg CO_2_-C g^-1^ hr^-1^	28.8 (2.1)	28.1 (1.2)	30.9 (1.2)	29.7 (1.1)

* Further details on the measurement of these variables can be found in the methods

## Discussion

Despite the fact that these lodgepole pine stands were mature with homogenous canopies dominated by a single species there was still considerable fine-scale variation in the understory plant communities, with four plant community types identified. These plant community types were associated with fine-scale variation in both above- and below-ground properties; there was substantial heterogeneity in below-ground microbial community structure and function, resources (e.g., boron), environmental conditions (e.g., soil pH), and also above-ground properties (e.g., mean tree diameter). Our results suggest that this heterogeneity is associated with variation in the organization and assembly of understory plant communities.

The most important above-ground variable associated with variation in understory plant community composition within these lodgepole pine forests was mean tree diameter. This was unexpected as a study in near boreal pine forests in eastern North America showed that fine-scale plant community composition varied along light gradients [22]. Thus we had hypothesized that canopy cover, which is often used as a surrogate for light transmission, would be an important above-ground variable. Our study forests generally had moderate canopy cover (see Table 1), and transmission of light to the forest floor was high enough to allow for presence of shade-intolerant pioneer species, such as *Chamerion angustifolium*, while some shade-tolerant species such as *Aralia nudicaulis* were also abundant; thus the lack of influence of canopy cover was not due to a lack of variability in light transmission levels. Litter cover was also associated with variation in the understory plant community (although not significantly different among plant community types). Previous studies have shown that litter cover affected bryophyte assemblages and was inversely related to bryophyte cover [67]. However, our finding that a particular community type (e.g., community type 3) had lower litter cover doesn’t necessarily imply inhibition by litter in the other community types. This result could simply be due to reduced litter production in quadrats of community type 3, as compared to the other types which had multi-layered understories including forbs, shrubs, and/or graminoids.

The eight below-ground variables that were significantly associated with fine-scale variation in understory plant community composition included pH, plant available boron, microbial respiration of malic acid, and abundance of five microbial PLFAs (14:0, a15:0, 16:1 2OH, cy17:0, 18:1ω7c). The microbial community is impacted by the soil pH, and it is generally accepted that fungi are favored over bacteria at low pH [68]. This pattern has been shown across large pH ranges [69], but also with narrow ranges of soil pH such as we observed [70]. The different PLFAs we observed at higher pH (bacterial PLFAs 18:1ω7c, a15:0 and cy17:0 and PLFA 16:1 2OH) as compared to the bacterial PLFA 14:0 that was associated with lower pH, suggest that heterogeneity in soil pH in these forests is associated with heterogeneity in soil microbial community composition. This fine-scale heterogeneity in pH and the microbial community composition, in turn, will influence biogeochemical cycles in these forests; for example, it could affect the soil concentrations of plant root exudates (including malic acid, which is a root-derived organic carboxylic acid [71]) and plant available boron. Boron is an essential micronutrient for higher plants, playing an important role in the formation and structure of plant cell wall complexes [72]; boron deficiency is known to inhibit plant growth [73]. Boron adsorbs to the soil at high pH levels and this has been found to reduce its availability for plant uptake [74], but within the narrow acidic range of pH in this study we saw an increase in soil available boron associated with increasing pH and this was likely because boron is still highly mobile at the low pH levels found in our plots. Overall, our findings suggest that below-ground heterogeneity is associated with the existence of fine-scale variation in understory plant community composition within these forests. This below-ground heterogeneity could be an important driver of stand-level plant biodiversity.

Interestingly, nitrogen availability was not an important below-ground variable. Other studies have suggested that N is associated with the structuring of plant communities, including in other pine systems [22, 75]. There was substantial variation in N levels (ranged from 0–25.6 μg 10 cm^-2^ summer burial ^-1^), so the lack of relationship between variation in N availability and plant community composition was not a function of a lack of variability in N availability. The dominant tall shrub in our forests, *A*. *crispa*, is an important N-fixer that understory plants may rely on, especially in N-limited systems [76]. Given the patchy distribution of alder among the 104 subplots it was surprising that we did not find a relationship between soil available N and variation in understory plant community types in this N-limited system. However, the forest floor mosses *H*. *splendens* and *P*. *schreberi* are also N-fixers and can comprise a significant portion of the N-fixation within boreal forests [19]. Therefore, the contributions of N from these forest floor mosses may have balanced out N contributions of alder. Alternatively, it could be that other nutrients, such as boron, are more important in influencing understory community composition. While another study found linkages between nitrogen availability and vegetation and soil microbial communities in reclaimed soils in northern Alberta, their study also showed that available boron levels may be associated with seasonal changes in the soil microbial communities [77].

The cluster analysis suggested there were four relatively distinct understory plant assemblages at a fine scale within these lodgepole pine forests and the db-RDA ordination further supported this. However, differences among the four plant community types were largely in relative abundance (cover) of species. This suggests that these species have fairly broad tolerances to the range of variation in resource availability and environmental conditions within these forests. Still the association between fine-scale variation in understory communities and below-ground variables in particular suggests that differences in tolerance among species led to variation in relative abundances. Our findings are consistent with how species tolerances to availability of resources have been shown to influence boreal understory plant community composition [78].

Moss species were the most significant indicators for three of the four plant community types. Our findings are in contrast to a previous study that found no evidence of habitat partitioning among moss species, including three of the moss indicators in our study (*D*. *polysetum*, *P*. *schreberi* and *P*. *crista-castrensis*) across gradients of temperature, vapour pressure deficit (VPD), precipitation, litterfall, or light [79]. A more recent study in mature lodgepole pine forests in the Canadian Rocky Mountains found a general pattern of increasing relative abundance of forest floor mosses over time, but with most of the variation in composition associated with spatial rather than the environmental variables [80]. Our results suggest that the tree size, litter cover, below-ground microbial structural and functional traits, and nutrient properties we measured in this study may be more strongly associated with variation in relative abundance of different moss species than the primarily abiotic variables (e.g., temperature, VPD) measured in previous studies [79] or [80].

In conclusion, this study provides new insights into the properties and processes that influence fine-scale variation in plant community composition and highlights important associations between understory plant communities and resource heterogeneity, including the structure and function of the forest floor microbial community. Below-ground soil microbial heterogeneity, which in turn influences biogeochemical cycling, may play a key role in driving fine-scale plant biodiversity within forests where there is a fairly homogeneous canopy dominated by a single tree species. Our findings have important implications for managing plant biodiversity. One such application could be predicting the effects of changing disturbance regimes on understory plant community composition. The type and intensity of disturbance and its relative impacts on the canopy, forest floor, and associated below-ground properties and processes will play an important role in determining the future composition and biodiversity of understory plant community types in forests. For example, the shift from a fire-dominated disturbance regime in lodgepole pine forests to one that includes partial stand disturbance by mountain pine beetle that kills canopy trees without directly impacting the understory plant community has long-term implications for both the above- and below-ground ecological properties and processes and, in turn, for understory plant community composition. Future studies should continue to explore the critical linkages between below-ground microbial community and understory plant community composition.

## Supporting Information

S1 Fig Indicator species analysis selection of cluster groups. (DOCX)

S2 Fig Results of hierarchical agglomerative cluster analysis of understory quadrats showing the four plant community types (indicated by symbols at the bottom (left) of the dendrogram. (DOCX)

S1 File MS Excel file that contains the raw data that were used in the analysis in this study. (XLSX)

S1 Table List of species and taxa in the study and the number of quadrats (n) that they were observed in. (DOCX)

S2 Table List of environmental variables included in study analysis. (DOCX)
