# Supplementary material for: Understory Plant Community Composition Is Associated with Fine-Scale Above- and Below-Ground Resource Heterogeneity in Mature Lodgepole Pine (Pinus contorta) Forests
Source: PLoS One. 2016 Mar 14;11(3):e0151436. doi: 10.1371/journal.pone.0151436 (PMC4790852; doi:10.1371/journal.pone.0151436)
Supplement: S1 Fig — (DOCX) [file pone.0151436.s001.docx]

**S1 Fig.** Indicator species analysis for A) change in mean p-value from 5000 randomization tests using a range of 2-12 clusters for the 104 quadrats; and B) the number of significant indicators with P < 0.05 for each of 12 steps of clustering.
