# Supplementary material for: Understory Plant Community Composition Is Associated with Fine-Scale Above- and Below-Ground Resource Heterogeneity in Mature Lodgepole Pine (Pinus contorta) Forests
Source: PLoS One. 2016 Mar 14;11(3):e0151436. doi: 10.1371/journal.pone.0151436 (PMC4790852; doi:10.1371/journal.pone.0151436)
Supplement: S2 Fig — (DOCX) [file pone.0151436.s002.docx]

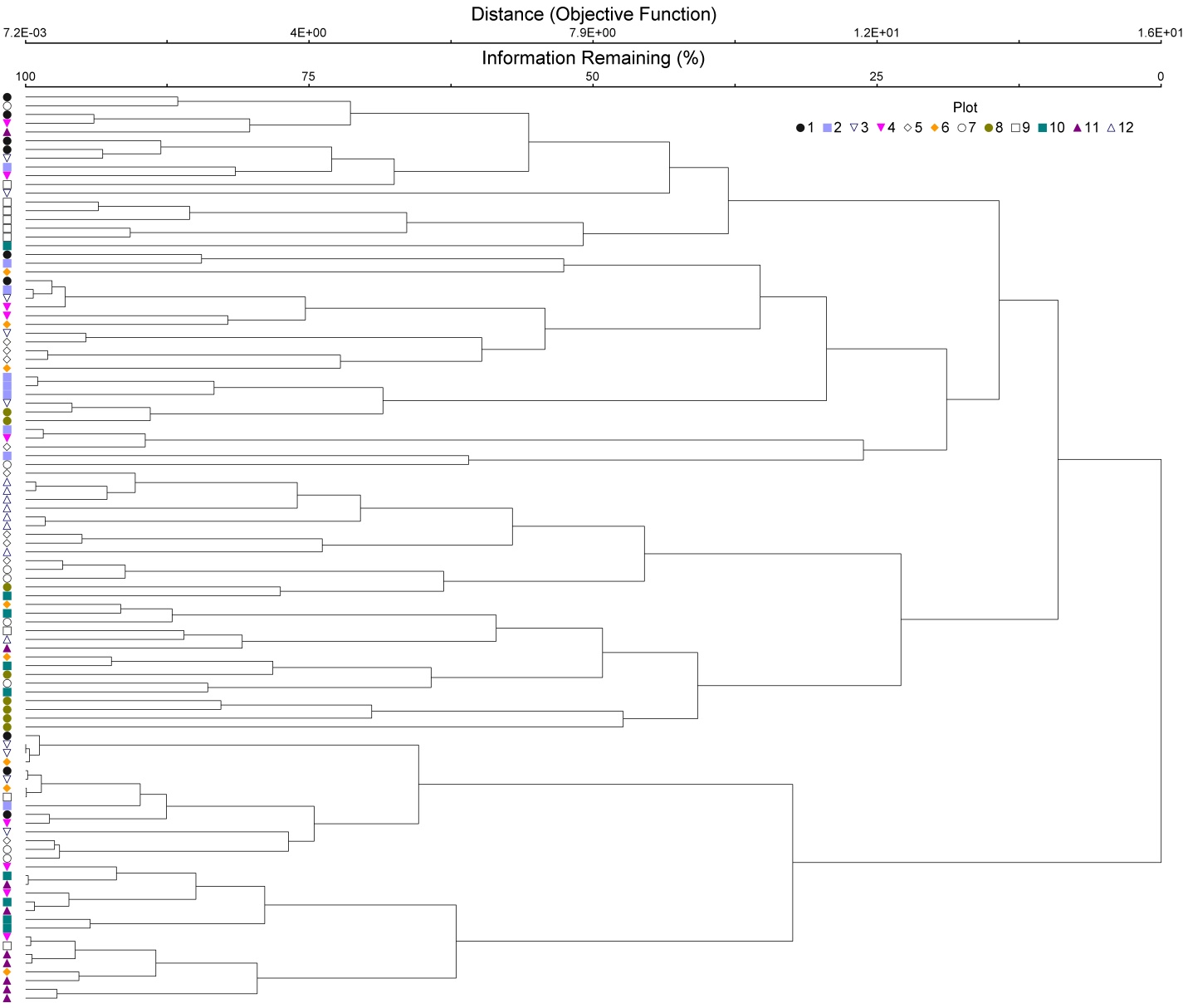


CT 4

CT 3

CT 2

CT 1

**S2 Fig**. Results of hierarchical agglomerative cluster analysis of understory quadrats showing the four plant community types (indicated by symbols at the bottom (left) of the dendrogram. Cluster analysis used a flexible beta linkage method with β= -0.25 and Sørenson’s distance measure. Chaining = 1.26%. The symbol used to identify each quadrat is the plot (1-12) it was located in. A vertical line indicates the separation of the four plant community types (CT) and which community type is associated with each cluster.
