# Supplementary material for: Understory Plant Community Composition Is Associated with Fine-Scale Above- and Below-Ground Resource Heterogeneity in Mature Lodgepole Pine (Pinus contorta) Forests
Source: PLoS One. 2016 Mar 14;11(3):e0151436. doi: 10.1371/journal.pone.0151436 (PMC4790852; doi:10.1371/journal.pone.0151436)
Supplement: S1 Table — (DOCX) [file pone.0151436.s004.docx]

S1 Table. List of vascular and non-vascular plants and lichens sampled in the study and the number of quadrats (n=108) in which each taxa was found. The 24 common species are highlighted in bold. Nomenclature follows the USDA Plants database (<http://plants.usda.gov/>).

| **Code*** | **Genus** | **Species** | **n** | **Scientific authority** | **Growth form** |
| --- | --- | --- | --- | --- | --- |
| ARCO | *Arnica* | *cordifolia* | 3 | Hook. | forb |
| **ARNU** | ***Aralia*** | ***nudicaulis*** | **31** | **L.** | **forb** |
| BRST | *Brachythecium* | *starkei* | 1 | (Brid.) Schimp. | bryophyte |
| **CACA** | ***Calamagrostis*** | ***canadensis*** | **8** | **(Michx.) Beauv.** | **graminoid** |
| **CAMO**† | ***Calamagrostis*** | ***montanensis*** | **51** | **Scribn. ex Vasey** | **graminoid** |
| **CHAN** | ***Chamerion*** | ***angustifolium ssp angustifolium*** | **60** | **(L.) Holub** | **forb** |
| CILA | *Cinna* | *latifolia* | 2 | (Trevir. ex Göpp.) Griseb. | graminoid |
| **COCA** | ***Cornus*** | ***canadensis*** | **104** | **L.** | **forb** |
| **DIPO** | ***Dicranum*** | ***polysetum*** | **33** | **Sw.** | **bryophyte** |
| DRAU | *Dryopteris* | *austriaca* | 1 | (Jacq.) Woynar ex Schinz & Thellung | fern |
| ELIN | *Elymus* | *Innovatus* | 2 | (Beal) Pilg. | graminoid |
| EQSY | *Equisetum* | *sylvaticum* | 2 | L. | forb |
| GOOB | *Goodyera* | *oblongifolia* | 1 | Raf. | forb |
| **HYSP** | ***Hylocomium*** | ***splendens*** | **67** | **(Hedw.) Schimp.** | **bryophyte** |
| **LIBO** | ***Linnaea*** | ***borealis*** | **97** | **L.** | **forb** |
| LICO | *Listera* | *cordata* | 4 | (L.) R. Br. | forb |
| LOIN | *Lonicera* | *involucrata* | 3 | (Richards.) Banks ex Spreng. | shrub |
| **LYAN** | ***Lycopodium*** | ***annotinum*** | **34** | **L.** | **club-moss** |
| LYCL | *Lycopodium* | *clavatum* | 1 | L. | club-moss |
| LYCO | *Lycopodium* | *complanatum* | 1 | L. | club-moss |
| **MACA** | ***Maianthemum*** | ***canadense*** | **55** | **Desf.** | **forb** |
| MEPA | *Mertensia* | *paniculata* | 1 | (Ait.) G. Don | forb |
| MINU | *Mitella* | *nuda* | 1 | L. | forb |
| **PEAP** | ***Peltigera*** | ***aphthosa*** | **12** | **(L.) Willd.** | **lichen** |
| **PEPA** | ***Petasites*** | ***palmatus*** | **9** | **(Aiton) A. Gray** | **forb** |
|  |  |  |  |  |  |
| **PLSC** | ***Pleurozium*** | ***schreberi*** | **99** | **(Brid.) Mitt.** | **bryophyte** |
| **POCO** | ***Polytrichum*** | ***commune*** | **52** | **Hedw.** | **bryophyte** |
| **PTCR** | ***Ptilium*** | ***crista-castrensis*** | **82** | **(Hedw.) De Not.** | **bryophyte** |
| **PYAS** | ***Pyrola*** | ***asarifolia*** | **8** | **Michx.** | forb |
| PYSE | *Pyrola* | *secunda* | 3 | L. | forb |
| **RHGR** | ***Rhododendron*** | ***groenlandicum*** | **16** | **(Oeder) Kron & Judd** | **shrub** |
| **ROAC** | ***Rosa*** | ***acicularis*** | **75** | **Lindl.** | **shrub** |
| RUAC | *Rubus* | *aculiferus* | 1 | L. | shrub |
| RUPE | *Rubus* | *pedatus* | 2 | J. E. Smith | shrub |
| **RUPU** | ***Rubus*** | ***pubescens*** | **24** | **Raf.** | **shrub** |
| **SPBE** | ***Spirea*** | ***betulifolia*** | **11** | **Pallas** | **shrub** |
| TRHY | *Trifolium* | *hybridum* | 2 | L. | forb |
| TRPR | *Trifolium* | *pratense* | 1 | L. | forb |
| **VAspp.** ‡ | ***Vaccinium*** | ***caespitosum and myrtilloides*** | **58** | **Michx.** | **shrub** |
| **VAVI** | ***Vaccinium*** | ***vitis-idaea*** | **49** | **L.** | **shrub** |
| **VIED** | ***Viburnum*** | ***edule*** | **26** | **(Michx.) Raf.** | **shrub** |
| **VIRE** | ***Viola*** | ***renifolia*** | **16** | **A. Gray** | forb |

*The 24 taxa that were used in the community analyses are highlighted in bold.

† CAMO did not flower in our sites, so a small number of individuals may have been misidentified as CAMO that were in fact other graminoid species, so in the manuscript we have referred to this as CAspp.

‡ *Vaccinium caespitosum* and *Vaccinium myrtilloides* were combined for analysis because they were only identified to genus in the field.
