# Supplementary material for: Understory Plant Community Composition Is Associated with Fine-Scale Above- and Below-Ground Resource Heterogeneity in Mature Lodgepole Pine (Pinus contorta) Forests
Source: PLoS One. 2016 Mar 14;11(3):e0151436. doi: 10.1371/journal.pone.0151436 (PMC4790852; doi:10.1371/journal.pone.0151436)
Supplement: S2 Table — (DOCX) [file pone.0151436.s005.docx]

**S2 Table**. List of blocking and above- and below-ground variables that were included as potential explanatory variables for the distance-based redundancy analysis, with units (if appropriate), and additional descriptors that provide more detail (if appropriate).

| **Variable** | **Units** | **Additional descriptors** |
| --- | --- | --- |
| Plot | n/a | Blocking factor - 1-12 (plots 1-4 study unit 1, 5-8 in study unit 2, 9-12 in study unit 3) |
| *Above-ground variables* | | |
| Basal area | m^2^/ha | Tall shrubs; Trees (Total and by live/dead status) |
| Cover | % | Canopy; Litter; Mineral Soil; Rock; Wood |
| Diameter at breast height (dbh) | cm | Total and by live/dead status |
| Downed woody material (DWM) | Mg ha^-1^ | By size class (0-0.5 cm, 0.5-1.0 cm, 1-3 cm, 3-5 cm, 5-7 cm and > 7 cm) |
| Stem density | trees ha^-1^ | Trees (by species and by live status) |
| *Below-ground variables* | | |
| Catabolic evenness | n/a |  |
| Decomposition | % |  |
| FH depth | mm |  |
| Fungi:bacteria PLFA ratio | n/a |  |
| Metabolic quotient | µg CO_2_-C g^-1^ hr^-1^ g^-1^ |  |
| Nutrient availability | µg -10 cm^2^ -burial length^-1^ | Al, B, Ca, Fe, K, Mg, Mn, NH_4_^+^, Total N, P, S, and Zn |
| pH | n/a |  |
| PLFA biomass | g | Actinomycetes, Arbuscular Mycorrhizal, Bacterial, Fungal, Total |
| PLFA | mol-% | There were 54 PLFAs used in the analysis |
| Substrate-induced respiration (MSIR) | µg CO_2_-C g^-1^ hr^-1^ |  |
